# BAIT: A New Medical Decision Support Technology Based on Discrete Choice Theory

**DOI:** 10.1177/0272989X211001320

**Published:** 2021-03-30

**Authors:** Annebel ten Broeke, Jan Hulscher, Nicolaas Heyning, Elisabeth Kooi, Caspar Chorus

**Affiliations:** Councyl, Delft, Netherlands; Department of Surgery, Division of Pediatric Surgery, University of Groningen, University Medical Center Groningen, Groningen, Netherlands; Councyl, Delft, Netherlands; University of Groningen, University Medical Center Groningen, Beatrix Kinder Ziekenhuis, Division of Neonatology, Groningen, Netherlands; Councyl, Delft, Netherlands; Faculty Technology Policy and Management, Department of Engineering Systems and Services, Delft University of Technology, Delft, Netherlands

**Keywords:** decision aids, decision models, decision support systems, decision support techniques, end-of-life decision, necrotizing enterocolitis

## Abstract

We present a novel way to codify medical expertise and to make it available to support medical decision making. Our approach is based on econometric techniques (known as conjoint analysis or discrete choice theory) developed to analyze and forecast consumer or patient behavior; we reconceptualize these techniques and put them to use to generate an explainable, tractable decision support system for medical experts. The approach works as follows: using choice experiments containing systematically composed hypothetical choice scenarios, we collect a set of expert decisions. Then we use those decisions to estimate the weights that experts implicitly assign to various decision factors. The resulting choice model is able to generate a probabilistic assessment for real-life decision situations, in combination with an explanation of which factors led to the assessment. The approach has several advantages, but also potential limitations, compared to rule-based methods and machine learning techniques. We illustrate the choice model approach to support medical decision making by applying it in the context of the difficult choice to proceed to surgery v. comfort care for a critically ill neonate.

Medical decision making is characterized by a high degree of complexity, uncertainty, and time pressure. Many decisions also entail ethical dilemmas. As a consequence, a variety of medical decision support systems have been developed.^1–5^ These can be classified into knowledge-based and non-knowledge-based systems.^6^ The former require that experts perform the very difficult task of explicating their tacit knowledge into deterministic rules; furthermore, such rule-based systems struggle with capturing the subtleties that are present in real-life contexts. Non-knowledge-based systems require vast amounts of historical data, on which machine learning models are trained to extract implicit relations; these models are opaque, hampering interpretability and accountability.

We present a third way to capture and codify medical expertise (which we colloquially define here as “knowing what to do in a certain situation, and being able to explain why”) and to make it available to support medical decision making. Our approach, called Behavioral Artificial Intelligence Technology (BAIT), uses choice analysis techniques traditionally employed to identify preferences of large groups of consumers, citizens, or patients and to make predictions regarding their future choice behavior.^7–9^ We reconceptualize these econometric techniques and put them into practice for codifying the expertise of small groups of experts and supporting their decision making. The objective of BAIT is to make accessible to an expert or group of experts the combined expertise of their peers in the context of a particular decision problem. To illustrate the workings of BAIT, we focus on one of the most difficult (moral) choices in medicine: to proceed to surgery v. comfort care for a critically ill neonate.^10,11^

## Methods

### How Does BAIT Work?

First, together with 2 to 4 experts, the expert decision is specified (e.g., perform surgery or not in the context of a particular medical situation and patient profile), and factors are identified that presumably play a role in making that expert decision (e.g., gestational age). Then, for each factor, relevant ranges are determined indicating minimum and maximum values of the factor-values (e.g., 24–30 weeks for gestational age). Constraints are specified to preclude combinations of factor-values that are impossible or highly unlikely to occur in real life. Note that some factors may require no additional investigator manipulation (gestational age, sex) while some would require a predetermined way of being defined (e.g., progress since birth pre–necrotizing enterocolitis [NEC]).

Second, the structure of the choice model is determined; for example, it is decided if nonlinear weights are to be accommodated (e.g., a decreasing or increasing marginal importance) and/or interaction effects (i.e., an additional positive or negative weight assigned to a particular combination of factor-values). Depending on the situation, different choice model types can be specified such as binary or multinomial, nested, or (panel) mixed logit models^12,13^ or models based on alternative behavioral theories such as regret-minimization or taboo tradeoff-aversion.^14,15^

Third, a choice experiment is designed and implemented, in which the group of experts is invited to make a series of hypothetical choices based on scenarios mimicking the real decision situation. Different types of choice experiments can be used,^16^ depending on the specificities of the decision. In our case, a so-called single conjoint is used, which asks respondents a yes/no question in the context of a specific patient-context profile. Another option could be a choice from a multinomial set of candidates (e.g., in the context of triage). Each scenario is specified in terms of a different combination of values taken from the prespecified decision-factors and ranges, taking into account relevant constraints. Crucially, using so-called efficient design techniques, scenarios are constructed such that each choice generates a maximum amount of information.^16^

Fourth, the observed choices are used to estimate the importance weights of all factors, including their signs (positive or negative) and any nonlinear curvatures (e.g., concavity or convexity), using maximum likelihood techniques.^12,13^ This process involves comparing the model predictions to the actual choices made by the experts. By iteratively adjusting the weights embedded in the model, increasingly accurate choice probability predictions are generated, until no further improvements can be made. The final model’s empirical performance is tested by means of various model fit metrics.

In a fifth step, results are presented back to the experts. Factor weights are visualized, showing how each factor contributes to the experts’ decisions in the experiment. In addition, the choice model equipped with the estimated weights is used to assess particular artificial choice situations, including cases that were not included in the choice experiment. Generated assessments take the form a probability statement—for example, “The probability that an expert that is randomly sampled from the expert group would recommend (to the patient’s parents) to perform surgery on a patient with this profile equals 18%.” In conjunction with the probabilistic assessment, color coding is used to highlight which factors had a positive or negative contribution to the assessment.

### Case: Whether or Not to Operate on a Premature Neonate with NEC

NEC is a devastating intestinal disease, mainly occurring in (very) preterm neonates.^17,18^ Due to improved survival of the most preterm infants, NEC incidence is rising.^19^ For some 30% to 40% of preterm infants with a diagnosis of NEC, emergency abdominal surgery is necessary. In these cases, children will succumb when surgery is withheld. However, perioperative mortality rates can reach 50%, and long-term morbidity, such as neurodevelopmental deficits and gastrointestinal complications, occurs in over 75%.^20^ Each case therefore presents the treating medical team as well as the parents with the dilemma of whether proceeding to surgery will still be in the child’s best interest.^18^ We focus on the moment where the clinician gives a final recommendation to parents. At this point, parents have developed a preference for surgery or comfort care (or they may be in doubt).

This study was waived for ethical approval by the university medical centre groningen (UMCG) ethical board (METc 2020/245). Note that in the context of this article, the aim of this small-scale case study is to illustrate the workings of BAIT as a technical innovation, as opposed to presenting new insights into the decision making process of local medical professionals regarding their response to NEC cases.

## Results

**Figure 1:**
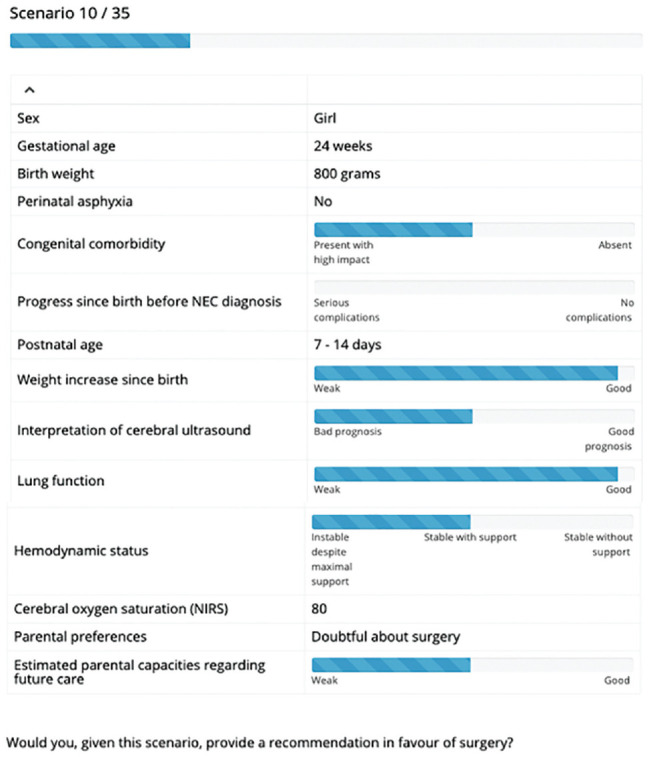
Example choice scenario.

Two pediatric surgeons and 2 neonatologists selected 14 factors with their ranges (see Table 1), which were subsequently combined into 35 choice scenarios (see Figure 1 for an example).

**Table 1:** Decision Criteria and Ranges

Factor	Level 1	Level 2	Level 3	Level 4
Gender	Boy	Girl		
Gestational age	24 wk	26 wk	28 wk	30 wk
Birth weight	500 g	650 g	800 g	1500 g
Perinatal asphyxia	Yes	Dubious	No	
Congenital comorbidity	Present with high impact	Present with minor impact	Absent	
Progress since birth before NEC diagnosis	Serious complications	Minor complications	No complications	
Postnatal age	0–7 d	7–14 d	14–21 d	
Weight increase since birth	Weak	Intermediate	Good	
Interpretation of cerebral ultrasound	Bad prognosis	Intermediate prognosis	Good prognosis	
Lung function	Weak	Intermediate	Good	
Hemodynamic status	Unstable despite maximal support	Stable with support	Stable without support	
Cerebral oxygen saturation (NIRS)	40	60	80	
Parental preferences	In favor of comfort care	Doubtful about surgery	In favor of surgery	
Estimated parental capacities regarding future care	Weak	Intermediate	Good	

NEC, necrotizing enterocolitis; NIRS, near infrared spectroscopy.

These were assessed by 15 experts (11 neonatologists and 4 surgeons). The estimated model obtained a good level of fit, as indicated by a McFadden’s ρ^2^ of 0.32. Choice probabilities predicted by the estimated model closely resemble the observed empirical relative frequencies: the mean absolute deviation equals 4.5 percentage points, implying that, for the average choice scenario, the predicted probability of a recommendation to operate was only 4.5 percentage points higher or lower than the observed relative frequency of choices made by the group of experts. Most factors turn out to have a linear effect on decisions, while some exhibit a nonlinear shape; weights are shown in Table 2.

**Table 2:** Estimation Factor Weights.^a^

Factor	Level	Weight (*P* Value)
Sex	Boy	0
	Girl	0.020 (0.96)
Gestational age	24 wk	0
	26 wk	1.656 (<0.001)
	28 wk	1.851 (<0.001)
	30 wk	2.859 (<0.001)
Birth weight	500 g	0
	650 g	1.238 (0.003)
	800 g	1.835 (<0.001)
	1500 g	2.507 (<0.001)
Perinatal asphyxia		0.452 (0.053)
Congenital comorbidity	Present with high impact	0
	Present with minor impact	0.944 (0.002)
	Absent	1.752 (<0.001)
Progress since birth before NEC diagnosis		0.230 (0.25)
Postnatal age		0.250 (0.28)
Weight increase since birth		0.183 (0.36)
Interpretation of cerebral ultrasound	Bad prognosis	0
	Intermediate prognosis	1.798 (<0.001)
	Good prognosis	2.782 (<0.001)
Lung function		0.204 (0.29)
Hemodynamic status		0.279 (0.144)
Cerebral oxygen saturation (NIRS)		0.430 (0.046)
Parental preferences	In favor of comfort care	0
	Doubtful about surgery	1.729 (<0.001)
	In favor of surgery	2.154 (<0.001)
Estimated parental capacities regarding future care		0.216 (0.28)
Constant		−8.830 (<0.001)

NEC, necrotizing enterocolitis; NIRS, near infrared spectroscopy. ^a^Decision: recommendation to operate (1) or not (0). Model: binary logit, estimated as binary logistic regression (using SPSS). Number of observations (N) = 525. Null log-likelihood = −364. Log-likelihood of estimated model = −245. McFadden’s ρ^2^ = 0.32.

**Figure 2:**
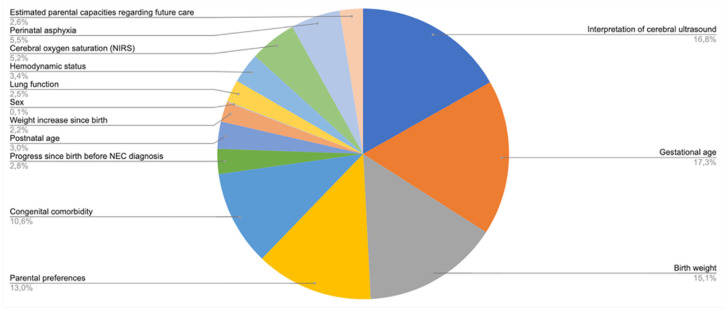
Relative importance of decision criteria.

**Figure 3:**
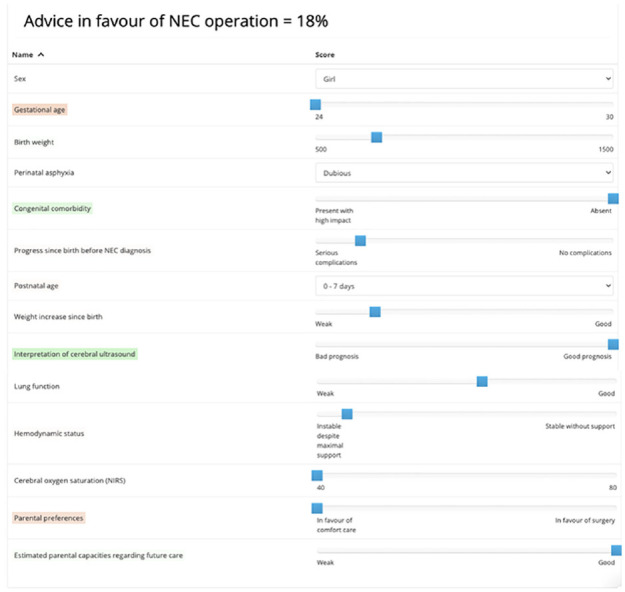
Example of an assessment generated by the model. Green color coding: the value for this factor contributed positively to the assessment. Red color coding: the value for this factor contributed negatively to the assessment. No/transparent color coding: the value for this factor did not contribute positively or negatively to the assessment.

As shown in Figure 2, 5 factors together make up three-quarters of the total importance of all factors combined. Parental preference, ranked fourth, makes up 13% of total importance. Figure 3 shows an example of an assessment generated by the model that was equipped with the estimated importance weights.

## Discussion

BAIT presents a clear alternative to conventional approaches. In contrast to rule-based or knowledge-based methods, BAIT uses choices to identify expertise rather than asking experts to explicate their expertise directly. This indirect process is aligned with the notion that humans find it very difficult to explain why they made a certain decision,^21^ especially when this involves moral judgment.^22^ Also, BAIT results in a flexible model that leads to choice probability predictions rather than attempting to capture the subtle tradeoffs of medical decision making into deterministic rules.

In contrast to machine learning models trained on historic data, BAIT offers a simple and explainable decision model: weights have an unambiguous meaning, and with help of color coding, it becomes clear immediately which combination of factors led to a particular decision. Furthermore, the choice experiment, being based on hypothetical scenarios, avoids data protection–related issues that may surface in the context of historic data.

In terms of a potential limitation of BAIT compared to rule-based expert systems such as clinical practice guidelines, we note that our approach relies on the ability of those experts who participate in the choice experiment, to assess, interpret, and balance a variety of factors and their potential risks for patient health and well-being. In light of the fact that even experienced medical experts may have difficulties assessing such risks,^23–25^ this may be considered a tall order. In a worst-case scenario, any misjudgment (or skew within the range of acceptable judgments) captured by BAIT in the choice experiment phase might carry over in the subsequent real-life decision making of experts, as their choices could be unduly influenced by the output of the decision support system. Indeed, more classical rule-based approaches such as the ones embedded in conventional guidelines and protocols may be considered less vulnerable to flaws and cognitive biases from the side of individual experts. We see several ways in which this potential problem of bias carryover can be reduced. First, the selection of experts participating in the choice experiment should be done very carefully; a tradeoff needs to be made here, between the need to select (only) experts with very high levels of expertise, while also ensuring that the group is large enough to avoid a situation where one expert’s misjudgment has an outsized effect on the model. Second, when the size of the pool of experts allows for this, it may be recommended to have experts perform the choice experiment in pairs (e.g., in our case, coupling a neonatologist to a surgeon), to allow for a discussion and balancing of opinions. Third, the very nature of BAIT suggests that in most contexts, it should best be used as a decision support system, as opposed to offering guidance.^26,27^ Concretely, BAIT is able to predict, in a given real-life choice situation, what decision would be made by which share of the expert pool. This, we expect, is very helpful to experts, but it does not equate to the use of a protocol or a set of rules and guidelines. In fact, such protocols and guidelines in our view can and should coexist with BAIT, the former being more prescriptive (focusing on what the individual expert “should” do) and the latter being more descriptive (focusing on what the pool of experts “would” do). Further research into the potential use of BAIT in real life is certainly recommended, to shed more light on this subtle distinction between decision guidance and decision support and how it affects expert decision making and learning in day-to-day medical practice.

Future research should focus on dynamic applications where the system is being updated with each new “real-life” choice made.

Another interesting avenue for further research is to study the transferability of expertise from one group of experts to another (e.g., in a different hospital). It should be noted here that our current application focused on a local and rather homogeneous group of experts; while this ensures that the resulting model is representative for the local situation, it does create potential risks of tunnel vision and bias carryover (see above). The application of BAIT on a larger scale (e.g., involving a choice experiment that is implemented among peers nationwide) could reduce such risks and hence deserves to be looked at in future studies.
